# RNA engineering approaches to aid high-resolution structure determination of medium-to large-sized RNAs

**DOI:** 10.1016/j.jbc.2026.113616

**Published:** 2026-09-30

**Authors:** Jerricho Tipo, Keerthi Gottipati, Marc C. Morais, Kyung H. Choi

**Affiliations:** 1 Department of Biochemistry and Molecular Biology, Sealy Center for Structural Biology, The University of Texas Medical Branch, Galveston, Texas, USA; 2 Department of Pharmacology and Toxicology, The University of Texas Medical Branch, Galveston, Texas, USA; 3 Department of Molecular and Cellular Biochemistry, Indiana University, Bloomington, Indiana, USA

**Keywords:** RNA structure, RNA engineering, crystallography, cryo-EM, tetraloop, tRNA scaffold, rRNA scaffold, pRNA scaffold, group I intron scaffold, group II intron scaffold

## Abstract

High-resolution structure determination of RNA remains a major challenge due to its conformational flexibility, intrinsic heterogeneity, and susceptibility to degradation. Although RNA plays central roles in gene regulation, viral replication, and cellular homeostasis, RNA-only structures represent a small fraction of entries in the Protein Data Bank. Overcoming the limitations associated with RNA has therefore required the development of innovative engineering strategies to stabilize defined conformations and facilitate structural analysis. In this review, we describe RNA engineering approaches that enable structure determination of medium- to large-sized RNAs by X-ray crystallography and cryo-EM. These advances include the use of stable tetraloops to promote proper folding and crystal packing, scaffold-based strategies employing tRNA and 5S rRNA for recombinant RNA production and stabilization, and bacteriophage Φ29 prohead RNA as well as group I and group II intron scaffolds to facilitate cryo-EM structure determination. Together, these approaches repurpose natural RNA structural features and host processing mechanisms into practical tools for RNA stabilization, large-scale production, and structural characterization.

RNA structure plays a central role in regulating cellular gene expression and function. Beyond serving as a linear template for protein synthesis, RNA adopts tertiary structures that determine its stability, localization, and interactions with proteins, other RNAs, and small molecules. Classic examples of structure-dependent RNAs include tRNA and rRNA, whose highly conserved folds are essential for accurate translation and ribosome function. Cellular mRNAs also contain functional structural elements, including iron-responsive elements in ferritin and transferrin receptor mRNAs, and internal ribosome entry sites in stress-response mRNAs that regulate translation and stability 1, 2. Viral RNAs similarly rely on complex structures to control replication, translation, and host interactions. For example, many viral genomes contain structured elements such as replication promoters that signal transition from viral translation to replication, packaging signals, frameshift-stimulating pseudoknots, and internal ribosome entry site elements that recruit ribosomes directly to the viral genome, enabling translation 3, 4, 5. Disruption of RNA structure is thus associated with numerous diseases, including viral infections and genetic disorders, highlighting the critical importance of understanding RNA folding.

Determining native RNA structure remains a significant bottleneck in understanding RNA function and its fundamental properties. Over 200,000 structures are available in the Protein Data Bank (PDB), yet only 3% (8150 depositions) include RNA and fewer than 1% (1890 depositions) represent RNA-only structures. RNA adopts a wide range of dynamic conformations owing to its highly charged phosphate-sugar backbone, the presence of a reactive 2′ hydroxyl group on the ribose sugar, and its ability to accommodate a plethora of stacking and folding interactions. The inherent heterogeneity in RNA structures arises from their ability to sample multiple low-energy conformational states, with structural flexibility often driven by transient and weak long-range interactions (6). These characteristics of RNA present unique challenges for structure determination using standard techniques such as NMR spectroscopy, X-ray crystallography, and cryo-EM. Choosing the appropriate method is critical. While NMR is particularly effective for probing the conformational dynamics of small RNAs and oligonucleotides, the technique becomes limited for medium to large RNAs (>50 nt) due to spectral overlap from the chemically similar RNA environment 7, 8. X-ray crystallography and cryo-EM are more suitable for medium to large RNAs. However, conformational heterogeneity and flexibility of RNA often limit resolution and data quality. In response to these challenges, innovative strategies have recently emerged to stabilize RNA conformations and enable high-resolution structural analysis.

This review highlights advances in RNA bioengineering and discusses innovative strategies for the rational design of stable RNA molecules, particularly medium- to large-sized RNAs. These strategies leverage intrinsic properties of RNA. First, we discuss the repurposing of RNA tetraloops to stabilize RNA hairpin ends. Tetraloops are naturally occurring, thermodynamically stable hairpin loops with defined folding preferences. Next, we discuss the use of stable cellular RNA scaffolds, such as tRNA and rRNA to support large-scale expression and structure determination of target RNAs. We then discuss the use of viral pRNA as a scaffold that directs the self-assembly of medium-sized RNAs into pentameric assemblies composed of five RNA molecules, making them well suited for cryo-EM structure determination. Finally, we describe group I and group II intron scaffolds that enable cryo-EM structure determination of target RNAs. We provide an overview of the design principles, practical considerations, and applications of each strategy.

## Stabilization of RNA stem-loops with tetraloop and pentaloop sequences

### Overview

A widely used strategy in designing RNA molecules amenable to structural studies is taking advantage of RNA tetraloops. Tetraloops are four-nucleotide, sequence-specific hairpin loops that cap double-stranded helices and reliably adopt well-defined, stable conformations. These loops are not only thermodynamically stable but also capable of transitioning from misfolded to correctly folded states 9, 10, 11, 12. By capping helical regions, tetraloops help nucleate and promote proper local folding, often improving the overall folding landscape of the RNA molecule (13). Thus, replacement of variable or poorly defined loop sequences with well-characterized tetraloops has become a common first-line approach for stabilizing RNA tertiary structures in biophysical and structural studies.

These stable RNA tetraloops were first observed in 16S rRNA, the RNA component of the small 30S ribosomal subunit in prokaryotes. The ∼1500 nt long 16S rRNA is highly structured and organized into four domains (I-IV) (14). Collectively, these domains contain 31 hairpin loop structures, 17 of which are tetraloops (Fig. 1*A*). Tetraloop sequences in 16S rRNA are commonly categorized into three classes: GNRA, UNCG, and CUYG, where “N” denotes any nucleotide (G, C, U, or A), “R” is a purine (G or A), and “Y” is a pyrimidine (C or U) (Table 1) 15, 16. Each tetraloop class adopts a unique and stable three-dimensional tetraloop geometry, stabilized through non-canonical base pairing, base stacking, and hydrogen bonding interactions involving the 2′ hydroxyl group of the ribose sugar (Fig. 1, *B–E*). For example, in GNRA tetraloops, G_1_ and A_4_ form a non-canonical base pair, while the N_2_, R_3_, and A_4_ form a base stack (Fig. 1*B*) 17, 18, 19. In UNCG tetraloops, U_1_ and G_4_ form a U⋅G wobble pair (a non-canonical base pair), while the C_3_ base stacks on U_1_ (Fig. 1*C*) 20, 21. In CUYG tetraloops, C_1_ and G_4_ form a canonical base pair with Y_3_, adopting variable conformation that may or may not stack on G_4_ (Fig. 1*D*) 22, 23. This variability of Y_3_ interaction makes the CUYG tetraloop more flexible than the structurally consistent GNRA and UNCG tetraloops. Extensive NMR structures have validated the structural integrity and solution stability of these tetraloops, underscoring their utility in RNA design 17, 19, 21.

**Figure 1:**
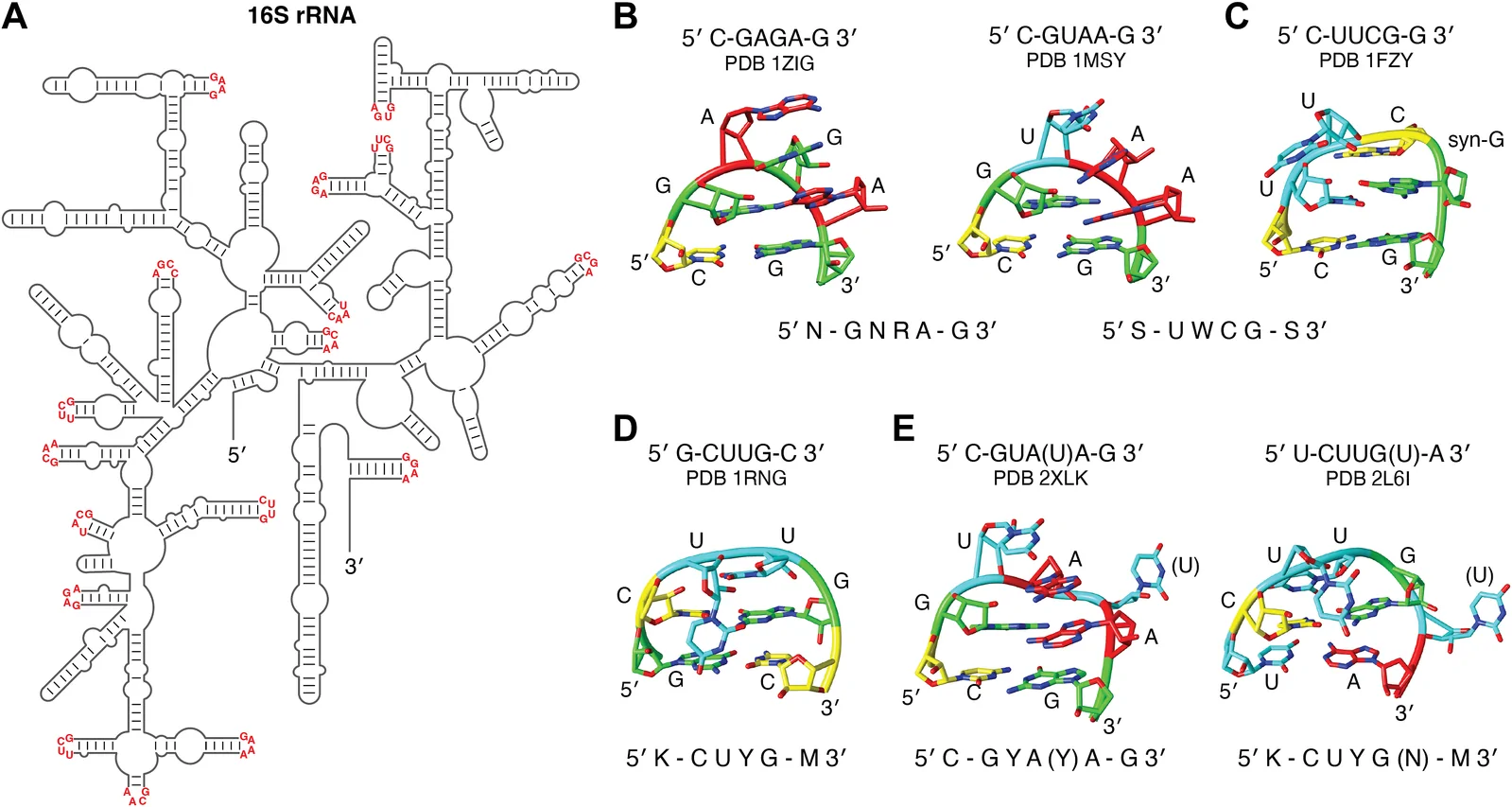
**Tetraloop structures and geometries.***A*, secondary structure of the 16S rRNA, which is enriched in tetraloops (highlighted in *red*). *B*, GNRA tetraloop. N and R represent any nucleotide and purine, respectively. The GAGA and GUAA tetraloops (PDB: 1ZIG and 1MSY, respectively) belong to the GNRA family and thus adopt nearly identical three-dimensional structures. *C*, UWCG tetraloop. W is either uracil or adenine. The UUCG tetraloop (PDB: 1FZY) is shown as a representative structure. *D*, CUYG tetraloop. Y represents a pyrimidine (cytosine or uracil). The CUUG tetraloop (PDB: 1RNG) is shown as an example. *E*, pentaloop sequences that adopt stable tetraloop-like conformations. The GYA(Y)A pentaloop (*left*, PDB: 2XLK) adopts a GNRA tetraloop-like fold, in which G_1_, U(Y)_2_, A_3_, and A_5_ fold as a GNRA tetraloop with U(Y)_4_ flipped out. The CUYG(N) pentaloop (*right*, PDB: 2L6I) adopts a CUYG-like structure, in which C_1_, U_2_, U(Y)_3_, and G_4_ form the CUYG tetraloop and U(N)_5_ is flipped out. K represents either guanine or uracil, and M represents cytosine or adenine. PDB, Protein Data Bank.

**Table 1:** Tetraloop and pentaloop sequence families

5′ nt	Tetraloop and pentaloop	3′ nt
N	GNRA	G
S	UWCG	S
K	CUYG	M
G	AUNA	C
K	UGGU	M
U	YGAR	G
Y	RMSA	R
C	GYAYA	G
K	CUYGN	M
K	GARYA	A
C	GCAAY	Y
C	SGCGA	G
G	MYKGR	C
G	SUSRU	C
G	UAAMK	C
U	USMMR	A
W	GAAAD	K
Y	UGUUC	G


Code	Nucleotide	
A	Adenine	
C	Cytosine	
G	Guanine	
U	Uracil	
R	Guanine/Adenine (purine)	
Y	Cytosine/Uracil (pyrimidine)	
K	Guanine/Uracil	
M	Adenine/Cytosine	
S	Guanine/Cytosine	
W	Adenine/Uracil	
B	Guanine/Uracil/Cytosine	
D	Guanine/Adenine/Uracil	
H	Adenine/Cytosine/Uracil	
V	Guanine/Cytosine/Adenine	
N	Adenine/Guanine/Cytosine/Uracil	

The stability of tetraloop structures is further demonstrated by the observation that certain pentaloops can adopt tetraloop-like geometries, provided that the characteristic tetraloop sequence and closing base pair are present 24, 25, 26. For example, GNRNA pentaloops (where N represents any nucleotide and R represents a purine) fold similarly to GNRA tetraloops, forming a non-canonical G_1_⋅A_5_ base pair and stabilizing the loop through stacking interactions between N_2_, R_3_, and A_5_ (Fig. 1*E*) (27). In addition, the identity and orientation of the closing base pair in the dsRNA stem immediately adjacent to the loop are critical in ensuring correct tetraloop folding (28). The GNRA and UNCG classes typically require closure by a 5′C-G3′ base pair, while CUYG tetraloops are stabilized by a 5′G-C3′ base pair (23). Since their initial discovery in rRNA, additional classes of tetraloops have been identified in a variety of biologically active RNAs, including self-splicing introns, viral genomes, and telomerase RNAs 29, 30, 31. Table 1 summarizes the tetraloop classes discussed here, along with other known tetraloop and pentaloop sequences identified across different RNA systems.

### Application of tetraloops in RNA structure determination

Tetraloops and pentaloops are valuable RNA engineering tools for structure determination using X-ray crystallography. They not only stabilize RNA hairpin termini but also promote crystal lattice formation through intermolecular base-pairing and stacking interactions 20, 32, 33. One common example is to engineer kissing-loop interactions, which naturally occur in biological systems between self-complementary tetraloops or between tetraloops and flexible single-stranded regions 34, 35. Hence, tetraloops can be strategically introduced in RNA stem-loops to facilitate stable crystal contacts. Below, we describe two representative examples, where tetraloop design facilitated crystallographic analysis.

In the first example, a GNRA tetraloop was used to facilitate the crystallization of 4.5S RNA, a component of the signal recognition particle (SRP), a ribonucleoprotein complex responsible for cotranslational targeting of proteins to membranes or for secretion (36). In *Escherichia coli*, the SRP comprises the Ffh protein and the 4.5S RNA. To obtain the crystal structure of this ribonucleoprotein complex, researchers used a minimized 49-nt version of 4.5S RNA, engineered with an optimized GNRA tetraloop from GGAA to GAAA. This 49-nt SRP RNA was then crystallized with the methionine-rich region of the Ffh protein (PDB: 1DUL). The resulting structure shows that the Ffh protein interacts along the minor groove of the RNA near the tetraloop (36). The GAAA tetraloop adopts the expected GNRA fold and mediates crystal contacts by interacting with the minor groove of a neighboring dsRNA helix, thus stabilizing the crystal lattice. A subsequent crystal structure of the ternary complex of SRP (4.5S RNA and Ffh protein) with its receptor FtsY, determined using the 4.5S RNA construct containing the native GGAA tetraloop, showed nearly identical overall SRP structure (PDB: 2XXA) (37). The two 4.5S RNAs superpose very well, including the tetraloop region, and exhibit highly similar protein-RNA interactions. Thus, optimization of tetraloop sequences can promote crystal lattice formation without altering the native RNA structure or its protein interactions.

The second example utilizes the conserved tertiary RNA-RNA interaction between the GAAA tetraloop and a tetraloop receptor (TR) RNA derived from self-splicing group I introns. In *Tetrahymena* *thermophila* group I introns, the apical GAAA tetraloop of stem P5 docks into the 11-nt bulged stem (TR) of P6, which stabilizes the active site and guides the splicing reaction (38). Importantly, this naturally occurring intra-molecular interaction can be engineered to promote intermolecular interactions in target RNA to facilitate crystal contacts (39). This strategy was successfully used to obtain crystals of the human hepatitis delta virus (HDV) ribozyme by inserting the GAAA loop and the TR motif into a stem that was not essential for the ribozyme’s biological activity (39). This method of replacing loop sequences with more stable tetraloops can also be integrated with other RNA-scaffold strategies discussed below (40).

### Considerations for the design and use of tetraloops

The tetraloop approach is particularly valuable for generating compact and stable RNA constructs and reducing conformational heterogeneity, while preserving the structural core. However, its successful application requires careful evaluation of the function of the native loop. Loops that participate in tertiary interactions, ligand recognition, protein binding, or catalytic activity are generally not suitable for substitution, as replacement may compromise the biological relevance of the resulting structure. In addition, the effect of tetraloop insertions is context-dependent, and different tetraloop classes may exhibit distinct interaction propensities depending on the overall structure of RNA. Consequently, a tetraloop that improves the behavior in one construct may not produce the same outcome in another. Selection of an appropriate tetraloop therefore may require empirical optimization.

## tRNA scaffold

### Overview

Structural and biochemical studies often require the synthesis of large quantities of RNA, commonly achieved through *in vitro* transcription using T7 RNA polymerase. While *in vitro* T7 transcription is a widely used and reliable approach, it presents several limitations for biochemical and structural studies. In particular, transcription termination by T7 RNA polymerase is not always precise and the enzyme frequently adds nontemplated nucleotides to the 3′ end of the transcript, resulting in heterogeneous populations. To improve 3′-end homogeneity, a DNA primer containing a 2′-O-methyl nucleotide at its terminus can be used to generate a transcription template (41). Alternatively, both the DNA primer and T7 RNA polymerase can be tethered to a solid support (42). However, neither approach is compatible with plasmid-based transcription templates. In addition, large-scale synthesis is constrained by the high cost of transcription reagents. To address these challenges, RNA engineering strategies have been developed in which the RNA of interest is fused to stable cellular RNAs, such as tRNA and 5S rRNA 43, 44, 45, 46. When expressed in bacterial cells, these fused RNAs are efficiently transcribed and protected from degradation, enabling high-yield RNA production and recovery. The use of tRNA and 5S rRNA scaffolds is discussed in the following two sections.

In the tRNA-scaffold approach, developed by Luc Ponchon and Frédéric Dardel, the target RNA is inserted into the tRNA anticodon stem, replacing the anticodon sequence, while leaving the remaining stems intact (Fig. 2, *A* and *B*) 43, 44. The DNA encoding the tRNA-fused RNA is cloned into the pBlueScript SK(+) plasmid for expression in *E. coli*. Transcription of the chimeric RNA is driven by the constitutive *lpp* promoter and terminated by the *rrnC* transcription terminator (44). Because a fully processed tRNA sequence containing a 3′ terminal CCA is used, no additional maturation steps are required to generate the tRNA chimera. The resulting tRNA-fused RNA is purified from *E. coli* lysates in a single step by anion exchange chromatography following phenol-chloroform extraction.

**Figure 2:**
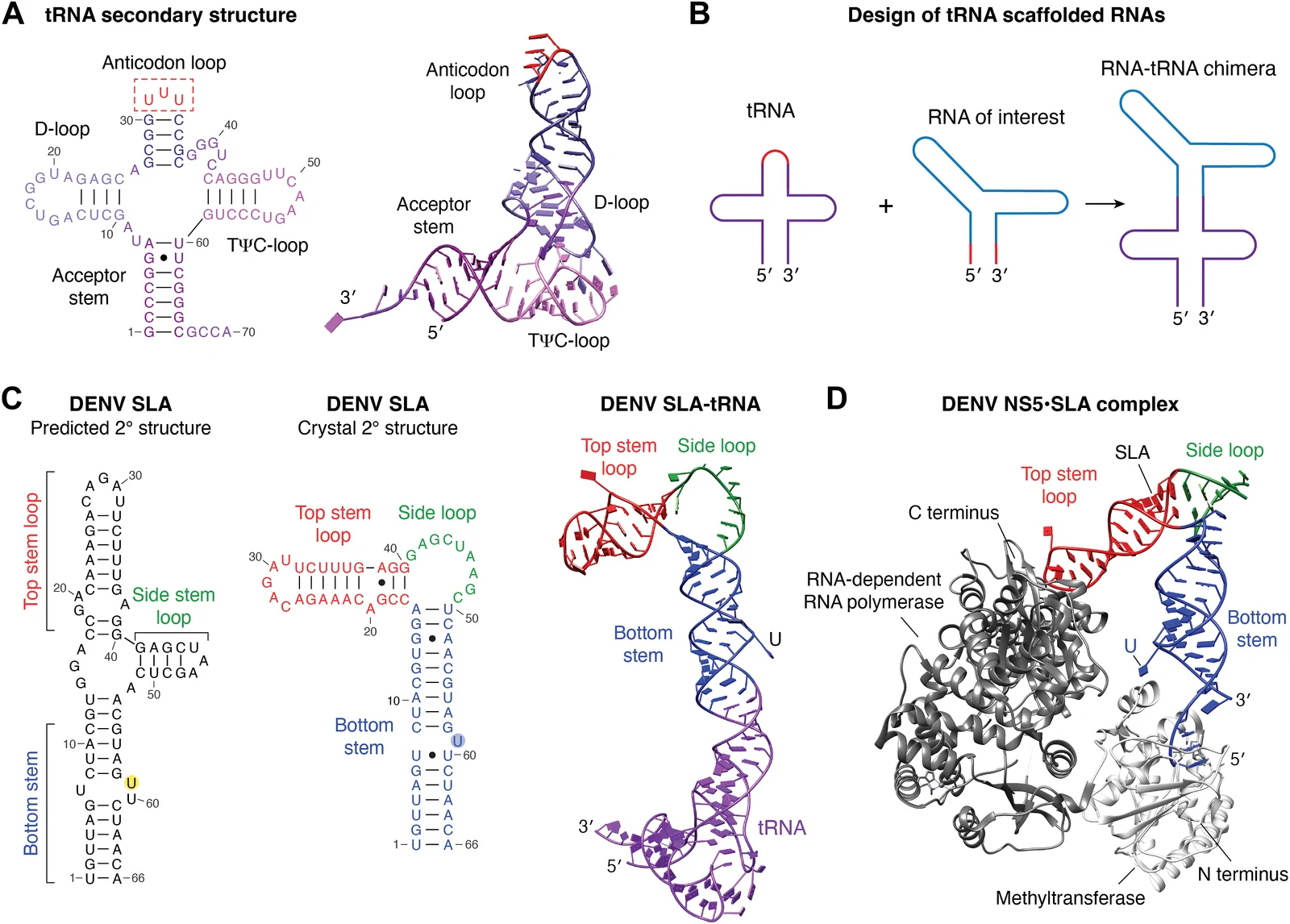
**The tRNA scaffold.***A*, structure of tRNA. The secondary structure of the tRNA^Lys^ consists of the acceptor stem (*dark magenta*), D-loop (*medium purple*), anticodon loop (*dark slate blue*), TΨC loop (*plum*), and CCA-3′ overhang (*purple*) (*left*). In the scaffold design, the anticodon loop (highlighted in *red*) is replaced with the RNA of interest. The tRNA adopts a compact L-shaped structure, stabilized by long-range interactions between the D and TΨC loops (PDB: 1EHZ) (*right*). *B*, design of tRNA-scaffolded RNAs. The anticodon loop (*red*) of tRNA (*purple*) is replaced with a double-stranded RNA region (*red*) from the RNA of interest (*blue*), generating an RNA-tRNA chimera that preserves the folds of tRNA and the inserted RNA element. *C*, secondary and tertiary structures of dengue virus stem-loop A (SLA). The predicted secondary structure of SLA consists of a bottom stem, a side stem-loop, and a top stem-loop, joined at a three-way junction (*left*). The crystal structure of dengue virus SLA determined using the tRNA-scaffold (PDB: 7LYF) reveals a bottom stem (*blue*), top stem-loop (*red*), and an open side loop (*green*) (*middle*). The SLA structure is shown with the tRNA-scaffold (*purple*). The side loop acts as a hinge to adjust the angle between the top and bottom stems (*right*). The bottom stem U-bulge, important for viral replication, is highlighted in the secondary structures. *D*, cryo-EM structure of the dengue virus NS5 and SLA replication complex. The NS5 protein consists of the N-terminal methyltransferase domain (*light gray*) and the C-terminal RNA-dependent RNA polymerase domain (*dark gray*). The dengue virus SLA forms an L-shaped fold that consists of a bottom stem, top stem-loop, and side loop and is colored as in (*C*) (PDB: 8GZP). The U-bulge interacts with the NS5 methyltransferase domain, facilitated by an adjustment in the angle formed at the SLA side loop. PDB, Protein Data Bank.

This approach offers several advantages for RNA production and structural studies. First, the tRNA scaffold eliminates the 3′-end heterogeneity commonly observed in RNAs generated by T7 *in vitro* transcription. Second, the tRNA scaffold limits conformational heterogeneity by preventing unwinding of RNA helices at the ends. Third, the resulting tRNA-fused RNA is larger than most endogenous tRNAs, allowing efficient purification from cellular RNAs by anion exchange chromatography. In addition, this method enables high-yield RNA production, as the chimeric RNA is constitutively transcribed from a plasmid. Under optimal conditions, 20 to 40 mg of purified tRNA-fused RNA can be obtained from a 1-L *E. coli* culture (47). The tRNA-scaffolded RNA constructs offer additional advantages for structure determination by X-ray crystallography. The tRNA domains can promote crystal lattice formation *via* intermolecular contacts with neighboring tRNA molecules. In addition, the conserved 3′-terminal CCA sequence can base pair with single-stranded regions of the target RNA, providing stabilizing crystal contacts. Finally, the tRNA scaffold can serve as a molecular replacement model, helping to overcome the phase problem and accelerate structure determination. Together, these features make the tRNA-scaffold approach both methodologically innovative and highly generalizable.

### Application of tRNA scaffold in structure determination

The tRNA-scaffold strategy has been used to express a wide range of structured RNAs, including those predicted to form stem-loops, three-way junctions, and four-way junctions, for structural and biochemical studies 47, 48, 49, 50, 51. Here, we describe the structure of the orthoflavivirus stem-loop A (SLA), which was determined using this tRNA scaffold approach. Orthoflaviviruses are enveloped, positive-sense RNA viruses that include several important human pathogens, such as West Nile virus, dengue virus, and Zika virus (52). Their genomes consist of a 5′ cap, a 5′ UTR, a single long ORF and a 3′ UTR (53). Viral genome replication and 5′ cap methylation are mediated by the multifunctional NS5 protein, which possesses both RNA-dependent RNA polymerase and methyltransferase activities 54, 55. Both enzymatic functions require its interaction with a noncoding RNA element located in the 5′ UTR, known as SLA 56, 57. Thus, this ∼70-nt RNA structure serves a dual role: it functions as an RNA promoter for negative strand synthesis and acts as a substrate for methylation of the 5′-cap of the genomic RNA.

The crystal structures of Zika and dengue virus SLA were determined using an SLA-tRNA chimeric design, in which the SLA sequences (nucleotides 1–70) were inserted into the anticodon loop between the 5′ and 3′ regions of the tRNA^Lys^ scaffold (48). To stabilize the chimeric RNA and generate a continuous dsRNA helix, two terminal nucleotides of the SLA were mutated to form Watson-Crick base pairs within the linker region between the tRNA^Lys^ scaffold and the SLA. Secondary structure predictions using the RNAfold server indicated that the RNA chimera preserves the same folds for individual tRNA and SLA elements. The resulting SLA-tRNA constructs retained functional interactions with the viral NS5 protein (48). Both SLA-tRNA chimeras crystallized; however, the initial Zika virus SLA crystals did not diffract to high resolution. To improve diffraction quality, the Zika SLA chimeric construct was further modified by introducing one or two additional base pairs in the linker region between the tRNA^Lys^ scaffold and the SLA. A single base pair insertion in the dsRNA helix results in an approximately 36° rotation of the stem, thereby promoting alternative crystal packing.

The tRNA scaffold was essential for crystallization, as the tRNA moiety participates directly in crystal contacts in both the dengue and Zika virus SLA structures. Based on secondary structure predictions, SLA was expected to adopt a three-way junction comprising a bottom stem, a top stem-loop, and a side stem-loop (48). The crystal structures of dengue and Zika SLAs showed deviations from secondary-structure predictions near the three-way junction, with the predicted side stem-loop instead adopting a single-stranded conformation that participates in intermolecular interactions (Fig. 2*C*) (48). The SLA-tRNA constructs were also used in mutational and biochemical studies, which demonstrated that SLA engages NS5 through the apical loop of the top stem-loop (58). These conclusions were later supported by a cryo-EM structure of the dengue virus NS5-SLA complex determined using RNA lacking the tRNA scaffold (Fig. 2*D*) (59). Thus, the tRNA-scaffolded RNAs retain the native structure and conserved features critical for biological function.

### Considerations for design and use of the tRNA scaffold

The tRNA-scaffold strategy is a powerful tool for expressing structured RNAs and determining their structures by X-ray crystallography and cryo-EM. In our experience, this method has enabled the expression of RNAs up to ∼300 nt in length. Using this approach, we have determined the structures of several viral RNAs, including dengue and Zika virus SLAs, coxsackie virus and poliovirus cloverleaf RNAs, the coxsackie virus cloverleaf-3C complex, and HIV-1 Rev-response element (RRE) stem-loops 44, 47, 48, 49, 60. Successful design and application of tRNA-scaffolded constructs require careful consideration of several factors. To preserve the structure and stability of the inserted RNA in bacterial cells, a double-stranded stem of the target RNA (instead of a single-stranded region) should be fused to the anticodon stem of the tRNA in the correct 5′ and 3′ orientation. Secondary structure elements such as large internal loops, bulges, or multiway junctions should be avoided at the fusion site. We have found that fusion to single-stranded regions often results in poor expression, likely due to instability and degradation of the resulting chimeric RNAs. The final fusion construct should be evaluated using secondary structure prediction tools with and without the tRNA scaffold to ensure that the scaffold does not disrupt the native conformation. To minimize functional interference, the tRNA should be fused to a non-essential stem region of the target RNA. A concern when using RNA scaffolds is the potential for unintended interactions or impairment of the target RNA’s biological activity. Therefore, it is essential to experimentally validate that the scaffolded RNA retains its native function. In terms of crystal structure determination, insertion or deletion of one or two base pairs at the tRNA junction could improve the diffraction quality of crystals. The tRNA-scaffold approach can also be combined with tetraloop modifications to promote crystal lattice formation.

## 5S rRNA scaffold

### Overview

Another RNA scaffolding strategy employs 5S rRNA, as described by Victor Stepanov and George Fox 45, 46. In this approach, the ∼120 nt 5S rRNA from *Vibrio proteolyticus* is used as a scaffold to which a target RNA is fused for structural studies. Similar to the tRNA-scaffold system, the 5S rRNA scaffold takes advantage of the host cell’s RNA processing machinery to enable high-level expression of precisely processed RNA products. Ribosomes are essential ribonucleoprotein complexes responsible for the translation of mRNA into proteins. In prokaryotes, the 70S ribosome comprises the small (30S) and large (50S) subunits (61). The 50S subunit includes the catalytically active 23S rRNA that coordinates peptide bond formation and the structural 5S rRNA that stabilizes the interaction between the 50S and 30S subunits to form the active 70S ribosome. Because of its vital role in ribosome biogenesis, 5S rRNA is highly conserved across all domains of life 62, 63. Importantly, although the mature ribosome contains rRNA components in equimolar ratios, many genomes encode more copies of the 5S rRNA gene than the 16S or 23S rRNA genes (64). In *E. coli*, for example, the *rrnD* operon contains an additional copy of the 5S rRNA gene. This genetic imbalance leads to an excess of free 5S rRNA in the cytoplasm. This excess production of free 5S rRNA, together with its inherent structural stability, makes it a viable scaffold for generating large quantities of chimeric RNAs in bacterial cells.

The ∼120 nt 5S rRNA forms a Y-shaped three-way junction, consisting of three domains, α, β, and γ, with the α and β domains forming the two arms of the “Y”, while the γ domain forms the straight leg (65). The α domain comprises helical region I, the β domain includes helical regions II and III separated by an internal loop B, and the γ domain contains helical regions IV and V, separated by an internal loop D (Fig. 3, *A* and *B*). To exploit 5S rRNA as a structural scaffold, the RNA must be stably expressed without being incorporated into the ribosome. To achieve this, a mutant 5S RNA from *V. proteolyticus* was used, in which helical region III in the β domain was deleted (66). This deletion prevents ribosome assembly, allowing the modified 5S rRNA to remain unincorporated and available in the cytoplasm. To facilitate insertion of target RNA sequences, the internal loop B within the β domain was further replaced with a *Bst*EII restriction site, enabling cloning of target RNAs into this region (Fig. 3*C*) (66). The resulting 5S rRNA-fused chimera can be recombinantly expressed in *E. coli* and efficiently purified using standard phenol-chloroform RNA extraction followed by anion exchange chromatography (45). These 5S rRNA-scaffolded RNAs are suitable for downstream structural and biochemical studies.

**Figure 3:**
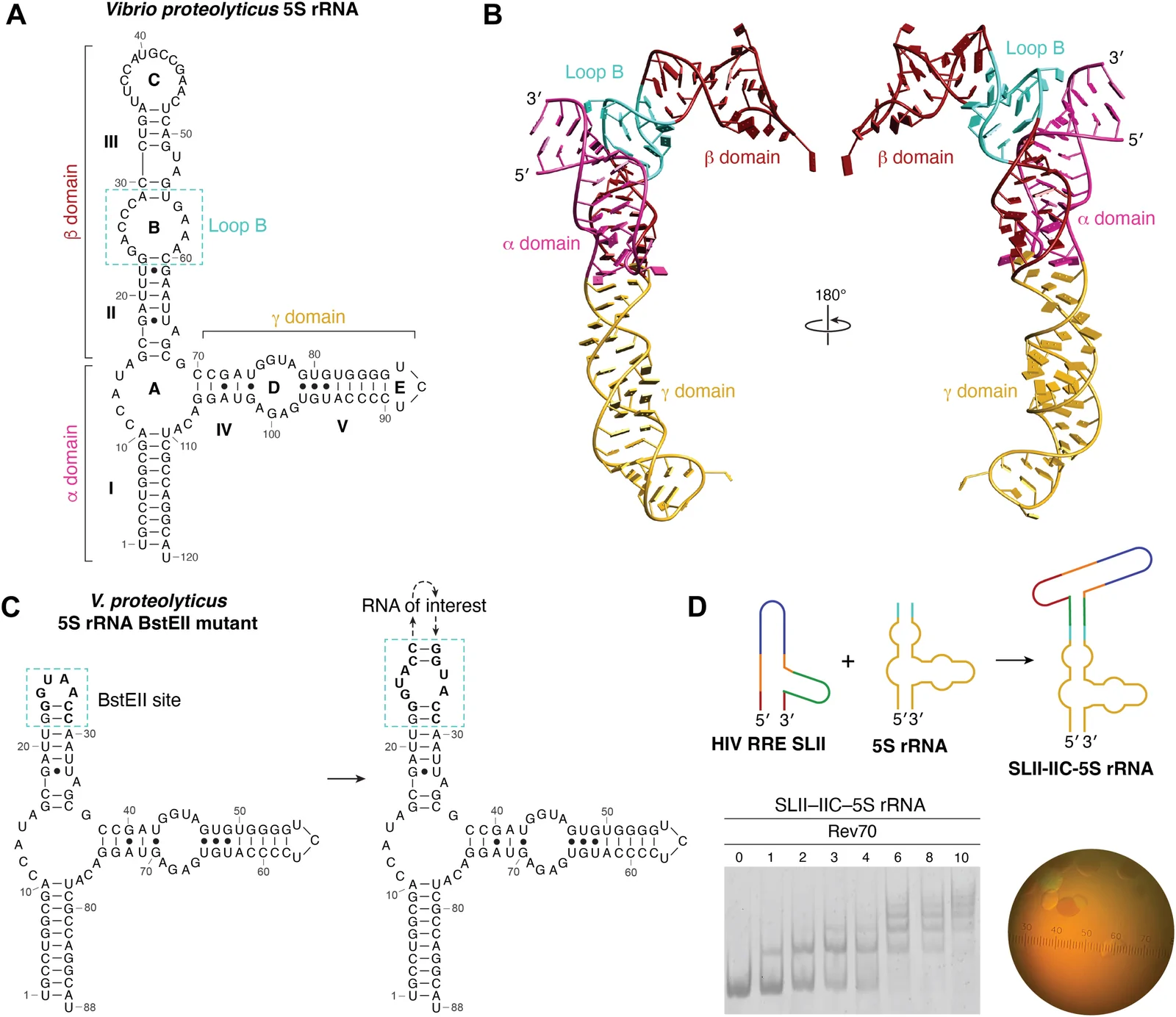
**The 5S rRNA scaffold.***A*, secondary structure of *Vibrio proteolyticus* 5S rRNA. The 120-nt 5S rRNA comprises five helical regions (I-V) and five substructures (*A–E*), organized into three domains, α, β, and γ. Loop B is highlighted with a *cyan dashed box*. *B*, tertiary structure of *Escherichia coli* 5S rRNA. The α domain (*magenta*), β domain (*dark red*), and γ domain (*yellow*) are connected by a three-way junction (loop A) (PDB: 6PJ6). The overall structure adopts a Y-shaped fold, with the α and β domains forming the two arms of the “Y” and the γ domain forming the stem. Loop B is colored in *cyan*. *C*, engineered 5S rRNA scaffold. Loop B (*cyan box*) of 5S RNA is replaced with the *Bst*EII restriction site (5′…G/GTNACC…3′), enabling insertion of an RNA of interest. This engineering generates an artificial loop B (*cyan box*) connected to the target RNA *via* a short dsRNA linker. *D*, application of the 5S rRNA scaffold. The scaffold was used to insert the HIV-1 rev response element stem-loop II, with stem-loop IIC (*green*) fused to the *Bst*EII site (*cyan*) (*top*). The RNA construct was validated biochemically *via* a gel shift assay with its binding partner, Rev70 protein (*bottom left*). Crystals of the 5S rRNA-scaffolded RNA were successfully obtained (*bottom right*).

### Application of 5S rRNA scaffold-assisted RNA for functional assays

Although high-resolution structures of RNAs fused to a 5S rRNA scaffold have not yet been reported, several studies have demonstrated that these chimeric RNAs retain their structure and function. For instance, ligand-binding activity of a malachite green-binding RNA aptamer (39 nt) was examined both in isolation and when fused to the 5S rRNA (67). Fluorescence-based binding assays showed that the RNA chimera and the non-scaffolded aptamer bind malachite green with similar dissociation constants (K_d_ of 0.9 and 1.0 μM for chimera and nonscaffolded aptamer, respectively) (67). These results indicate that the 5S rRNA scaffold does not interfere with binding.

We also used the 5S rRNA-scaffold strategy to express HIV-1 RNA elements such as the RRE and the 5′ UTR region. Both 5S rRNA-fused RNAs (162 and 194 nt) were stably expressed in *E. coli* and purified using phenol-chloroform extraction. Importantly, the 5S rRNA-fused RRE retained its ability to bind the HIV-1 Rev protein with an affinity comparable to that of the unmodified RRE, indicating that the 5S rRNA scaffold does not interfere with RNA function (Fig. 3*D*). In addition, crystals of 5S rRNA-fused RRE chimera could be obtained (Fig. 3*D*), although the structure was ultimately determined with a tRNA scaffold (49). The 5S rRNA-scaffolded 5′ UTR could also be visualized by cryo-EM, demonstrating the compatibility of this scaffold with multiple structural techniques.

### Considerations for the design and use of the 5S rRNA scaffold

The 5S rRNA scaffold is a valuable tool for the recombinant production of functional RNAs in large quantities, facilitating stabilization of the target RNA and enabling structural studies. When designing a 5S rRNA-scaffolded construct, many of the same considerations that apply to the tRNA scaffold should also be taken into account. Ideally, a double-stranded helical region of the target RNA should be fused into the 5S rRNA scaffold to minimize disruption of its function and preserve the predicted secondary structures of both the scaffold and the target RNA (Fig. 3*C*). As with any RNA fusion strategy, appropriate control experiments are essential to assess potential off-target effects introduced by the scaffold. These include testing both the isolated target RNA and the 5S rRNA-scaffolded RNA to ensure that the scaffold does not interact nonspecifically with the intended binding partners. If undesired interactions are observed, alternative scaffolds, such as tRNA or protein-based systems, should be considered.

## pRNA scaffold to mediate pentameric RNA assembly

### Overview

Bacteriophage Φ29 is a dsDNA virus that packages its genome into a preformed empty capsid, or prohead, using a powerful DNA-packaging motor (68). The motor assembles at a unique five-fold vertex of the prohead, where the connector proteins reside, and consists of a pentameric pRNA ring and a pentameric ATPase assembly (Fig. 4*A*) 69, 70. The 174-nt Φ29 pRNA comprises six helical regions (A-F), organized into two domains: Domain I (regions A and C-E) and Domain II (B and F) (Fig. 4*B*) (71). A 71-nt fragment within Domain I retains the structural elements required for assembly into the pentameric pRNA ring at the unique five-fold vertex of the prohead, and therefore serves as the minimal functional core for scaffold engineering (Fig. 4*C*) (72).

**Figure 4:**
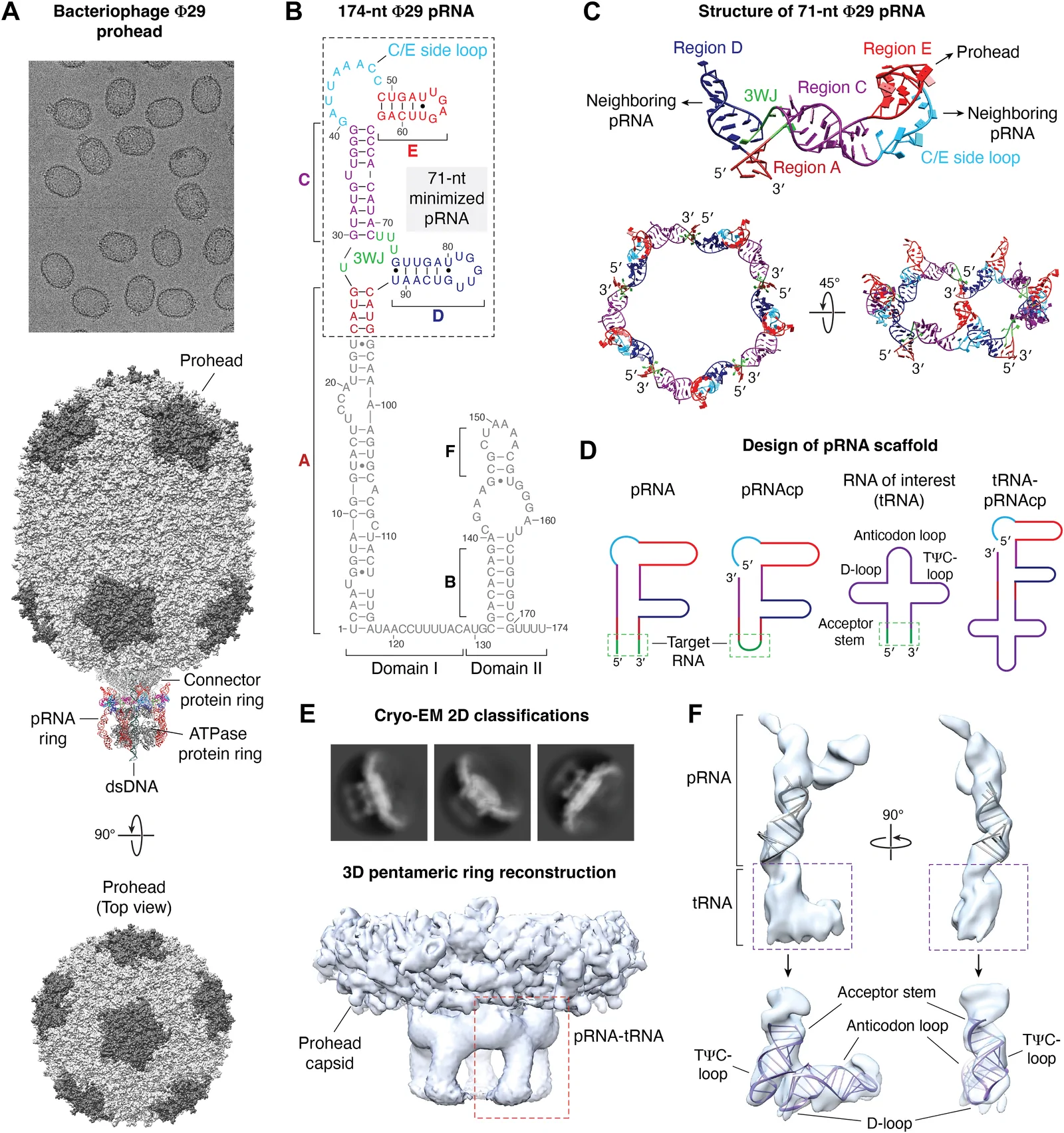
**The Φ29 bacteriophage pRNA scaffold for cryo-EM structure determination.***A*, Φ29 prohead and DNA packaging motor complex. A cryo-EM image of Φ29 proheads with assembled pRNA rings is shown (*top*). The side view of the prohead is shown with the motor components attached at the portal vertex, the connector protein (*light gray*), pRNA ring (*red*), and ATPase ring (*dark gray*), with dsDNA (*gray blue*) being packaged (*middle*). A top view of the prohead shows its five-fold symmetry (*bottom*). The structural model was generated using PDB: 6QVK and 7JQQ. *B*, secondary structure of the 174-nt Φ29 pRNA. The pRNA consists of six dsRNA regions (A-F) organized into two domains. Domain I contains regions A (*dark red*), C (*purple*), D (*dark blue*), and E (*red*). Regions A, C, and D are connected by a three-way junction (3WJ, *green*), while region E connects to C *via* a C/E side loop (*blue*). The minimized 71-nt pRNA sequence used as a scaffold is boxed. *C*, structure of the 71-nt pRNA. The pRNA assembles into a pentameric ring (PDB: 6QYZ). The pRNA monomer and pentamer are colored as in (*B*) (*top* and *bottom*, respectively). Assembly of pRNA requires interactions between region E and the major capsid protein subunits that circumscribe the connector vertex, as well as intermolecular pRNA contacts between the C/E side loop and D stem-loop. *D*, design of pRNA and circularly permuted pRNA (pRNA^cp^) scaffolds. In the pRNA^cp^ scaffold, region A is closed with a stem-loop, and new 5′ and 3′ ends are introduced within region C. For the pRNA^cp^-scaffolded tRNA construct, the tRNA acceptor stem is fused to region A of the pRNA^cp^ scaffold. *E*, cryo-EM structure determination of tRNA-pRNA^cp^. 2D class averages show pentameric pRNA rings assembled at the portal vertex (*top*). 3D reconstruction shows that the pRNA ring is attached to the prohead (*bottom*). *F*, cryo-EM structure of the tRNA-pRNA^cp^. The density corresponding to the tRNA-pRNA^CP^ was first isolated (*top*), revealing the fused tRNA extending downward from pRNA domain A, consistent with its position in the chimeric design. A second round of focused refinement on the tRNA region yielded a 4.8 Å structure, into which the tRNA model (*purple*) was fitted (*bottom*). pRNA, prohead RNA.

Two pRNA scaffold designs have been developed: one based on the WT pRNA sequence and the other on a circularly permuted pRNA (pRNA^cp^) (Fig. 4*D*) (73). In the WT scaffold, the native 5′ and 3′ termini provide convenient fusion sites for target RNAs. However, direct fusion at either terminus can introduce conformational flexibility because of increased rotational freedom between the scaffold and the fused RNA. To create a more rigid attachment site, the pRNA^cp^ scaffold was engineered by joining the native termini with a UAAA tetraloop and introducing new termini within stem C (Fig. 4*D*). This design preserves the structural elements required for assembly on proheads while providing a structurally constrained fusion site for target RNAs (74). This approach, termed the pRNA-assisted RNA Imaging Scheme (pARIS), enables stable presentation of target RNAs for structural analysis by cryo-EM.

### Application of pRNA-fused RNA construct

The pRNA^cp^ scaffold was used to determine the cryo-EM structure of a fused tRNA, yielding a 4.8 Å cryo-EM reconstruction of the tRNA (73). During 2D classification, additional density corresponding to the pentameric tRNA-pRNA^cp^ was readily observed at the unique five-fold connector vertex of the prohead (Fig. 4*E*). Cryo-EM processing begins with reconstruction of the entire prohead-pRNA complex, followed by reextraction of the pRNA region into a smaller box. Reconstruction imposing 5-fold symmetry clearly showed density corresponding to five tRNAs extending from the pentameric pRNA scaffold (Fig. 4*E*). Subsequent symmetry expansion and computational subtraction of four of the five pRNA copies generated a particle set corresponding to a single tRNA-pRNA^cp^, yielding a 5.3 Å reconstruction of the pRNA-tRNA that showed well-defined structural features of the scaffold (Fig. 4*F* top). Additional focused refinement using the tRNA as the alignment target produced a final 4.8 Å reconstruction, in which the canonical tRNA fold was clearly resolved and showed good agreement with the previously determined crystal structure (Fig. 4*F* bottom). These results demonstrate that pARIS substantially facilitates the cryo-EM analysis of small RNAs by increasing their effective molecular mass, enforcing a defined assembly geometry, and enabling symmetry expansion during image processing. Together, these features enable structure determination of RNA molecules that would otherwise be difficult to visualize because of their small size and conformational heterogeneity.

### Considerations for the design and use of pRNA scaffold

The pARIS approach offers several advantages for cryo-EM structure determination of small RNAs. The well-established Φ29 prohead system provides a streamlined sample preparation workflow. Attachment of pRNA-RNA chimeras to the prohead followed by vitrification is straightforward. Unlike isolated small RNAs, which often require extensive optimization of freezing conditions and ice thickness to allow particle identification, the large proheads are readily visualized in cryo-EM micrographs, reducing grid-screening time and increasing throughput. The pRNA scaffold also provides a built-in quality-control mechanism for correctly folded RNAs. Assembly onto the prohead requires preservation of the structural features necessary for pentamer formation and capsid binding. Consequently, misfolded or structurally compromised pRNA-fused RNAs are unlikely to assemble as pentamers on proheads, and are effectively excluded from cryo-EM analysis. This assembly-dependent recruitment enriches the sample for properly folded RNA molecules, improving compositional and conformational homogeneity. Proheads lacking assembled pRNA pentamers are easily filtered out at an early stage of processing.

Assembly of the pRNA scaffold on the prohead also improves cryo-EM image processing. The large well-defined structure of the Φ29 proheads provides a robust alignment reference and facilitates automatic particle picking by improving the signal-to-noise ratio of the attached RNA. In addition, Φ29 proheads generally exhibit a broad distribution of particle orientations, improving angular sampling of the attached RNAs during reconstruction 75, 76. In contrast, protein-free RNAs often exhibit preferred orientation on cryo-EM grids, with end-on views rarely observed (77). Finally, the pentameric geometry results in five copies of the RNA of interest per prohead particle, enabling symmetry expansion and focused refinement to substantially increase the number of analyzable RNA particles. Taken together, these features make pARIS a promising platform for extending cryo-EM structure determination to small and conformationally dynamic RNAs.

## Group I and II intron scaffold approaches in cryo-EM structure determination

### Overview

In principle, any stable, well-folded RNA can serve as a scaffold for the stabilization and structural analysis of target RNAs. Recent advances in cryo-EM have exploited highly structured RNAs, including group I and II introns, as scaffolds for the incorporation and stabilization of target RNAs 77, 78. Group I and group II introns are particularly well suited for this purpose because they are large (∼400 nt), autonomously folding RNAs that adopt compact, rigid tertiary structures stabilized by extensive long-range interactions (Fig. 5). Their modular architectures contain solvent-exposed peripheral helices that can accommodate insertion of target RNAs without disrupting the overall scaffold.

**Figure 5:**
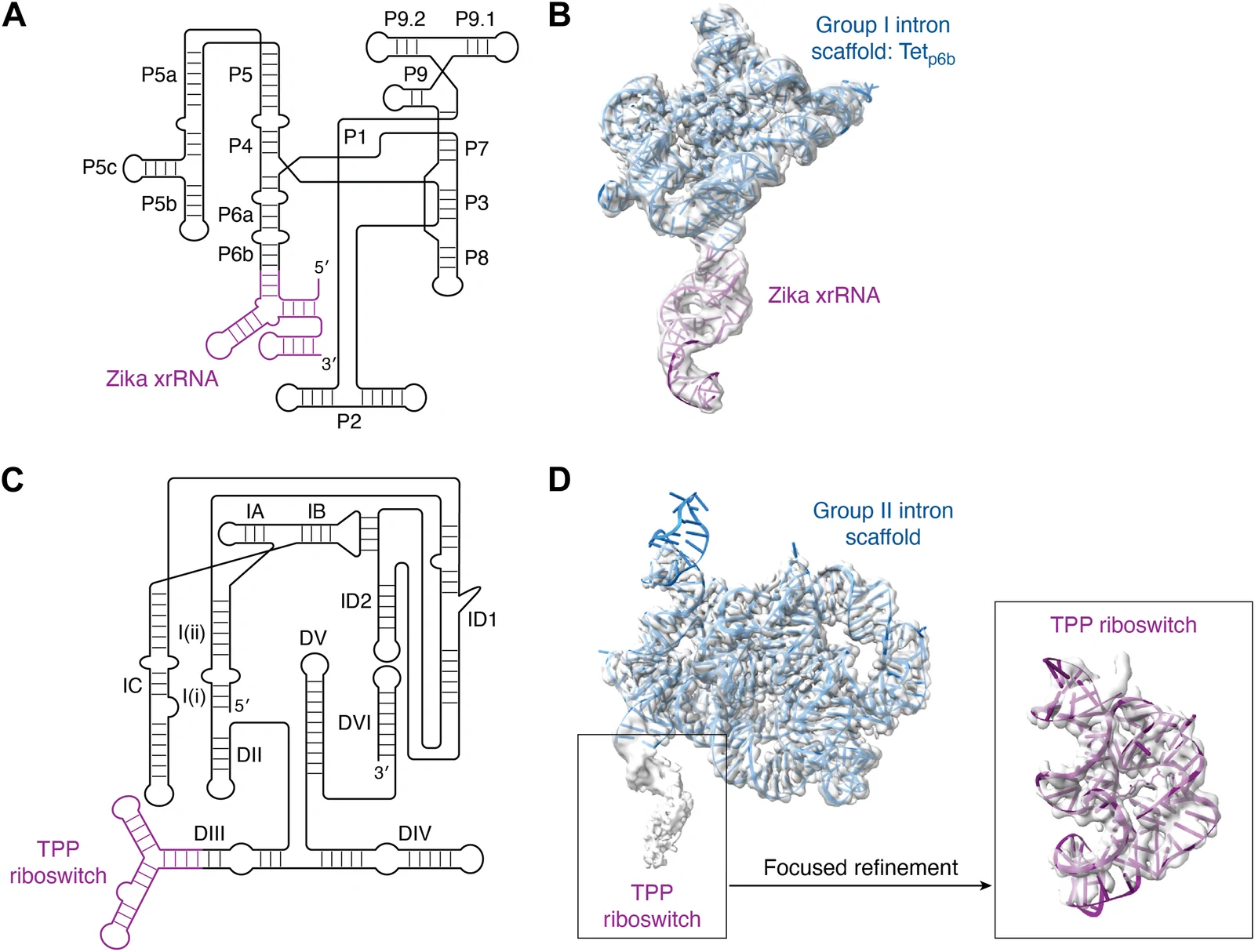
**Group I and group II intron-based RNA scaffolds for cryo-EM structure determination.***A*, secondary structure of the circularly permuted group I intron *Tetrahymena* ribozyme (Tet_P6b_) scaffold carrying the 23-kDa Zika virus xrRNA target RNA. The 5′ and 3′ ends of the circularly permuted Tet_P6b_ are connected to the xrRNA through an internal P6 stem-loop. The scaffold RNA is shown in *black*, and the target is shown in *purple*. *B*, cryo-EM map of the Tet_P6b_-scaffolded xrRNA construct (EMD: 41308) overlaid with the atomic model (PDB: 8TJQ). The overall resolution of the Tet_P6b_-xrRNA reconstruction is 3.4 Å, whereas the local resolution of the xrRNA is 5.0 Å. The Tet_P6b_ scaffold is shown in *blue* and Zika xrRNA is shown in *purple*. *C*, secondary structure of group IIC intron scaffold from *Oceanobacillus iheyensis* (*O.i*.) carrying the 28-kDa thiamine pyrophosphate (TPP) riboswitch target RNA. The target RNA is inserted into domain III of the group IIC intron scaffold, maintaining continuous 5′-3′ directionality. The scaffold RNA is shown in *black*, and the target RNA is shown in *purple*. *D*, cryo-EM structure of the group IIC intron-scaffolded TPP riboswitch. The 2.4 Å cryo-EM map of the scaffolded TPP riboswitch (EMD: 45248) is overlaid with the atomic model of the group IIC intron scaffold (PDB: 9C6J) (*left*). Focused refinement of the TPP riboswitch yielded a 3.1 Å reconstruction (EMD: 45249), shown with the corresponding atomic model (PDB: 9C6K) (inset). The group IIC intron scaffold and TPP riboswitch are shown in *blue* and *purple*, respectively. PDB, Protein Data Bank.

By rigidly coupling the target RNA to a well-defined structural framework, intron scaffolds increase the effective particle size, reduce conformational heterogeneity, and improve particle alignment during cryo-EM processing. In addition, the availability of high-resolution intron structures facilitates particle alignment and focused refinement during image processing, allowing the scaffold to serve as a structural anchor for visualization of the attached RNA. Collectively, group I and group II intron scaffolds enabled cryo-EM structure determination of RNAs ranging from 17 to 70 kDa at resolutions of 3 to 5 Å, demonstrating that compact, highly structured catalytic introns provide effective scaffolds for high-resolution cryo-EM analysis of RNAs 77, 78.

### Application of intron scaffold in cryo-EM structure determination

Langeberg and Kieft engineered the *Tetrahymena* group I intron ribozyme into an RNA scaffold for cryo-EM structure determination (78). The ∼400 nt *Tetrahymena* ribozyme is a self-splicing RNA composed of multiple paired (P) dsRNA regions, with the catalytic core spanning regions P3-7 (79). To repurpose the ribozyme as a scaffold, it was circularly permuted by joining the native 5′ and 3′ termini, and introducing new termini within the P6 stem while preserving the overall ribozyme structure. The newly created 5′ and 3′ termini were then fused to a stem of the target RNA, maintaining the correct 5′ to 3′ directionality. Using this strategy, the cryo-EM structures of Zika and Tamana bat virus xrRNAs (23 and 17 kDa, respectively) and a fluoride riboswitch (17 kDa) were determined at resolutions ranging from 4.5 to 5.0 Å (78). At these resolutions, the target RNAs could be modeled following placement of the known *Tetrahymena* ribozyme structure (Fig. 5, *A* and *B*).

Haack *et al.* recently developed a group II intron-based RNA scaffold for cryo-EM structure determination (77). Group II introns are self-splicing catalytic RNAs composed of six structural domains (Domains I-VI) that fold into a compact tertiary architecture. In this scaffold, the target RNA was inserted into Domain III, a solvent-exposed internal stem-loop of the ∼400-nt group IIC intron from *Oceanobacillus iheyensis* (*O.i*.), while preserving the native intron architecture (Fig. 5, *C* and *D*). Because group II introns are catalytically active, a catalytically inactive mutant was used to prevent cleavage of the scaffold or the inserted target RNA. As with the group I intron scaffold, a stem-loop region of the target RNA was fused directly to Domain III through a continuous helical junction that maintained the 5′-3′ orientation. Using this strategy, the structures of an 86-nt thiamine pyrophosphate riboswitch and a 210-nt *raiA* bacterial ncRNA were determined at resolutions of 3.1 and 3.0 Å, respectively (Fig. 5*D*) (77).

### Considerations for the design and use of group I and group II intron scaffolds

The successful application of group I and group II introns as cryo-EM scaffolds highlights several general principles for RNA scaffold design. An effective RNA scaffold should adopt a compact, rigid tertiary structure that increases the effective size of the target RNA while minimizing conformational flexibility and preferred particle orientation. The insertion site should be located within a solvent-exposed peripheral helix that can accommodate the target RNA without disrupting the native scaffold architecture. As with other RNA scaffold strategies, the target RNA should be fused through an existing helix rather than through flexible single-stranded linkers, as rigid helical junctions reduce conformational heterogeneity and improve particle alignment during cryo-EM image processing.

The group I and group II intron scaffolds differ in how the target RNA is incorporated. In the group I intron scaffold, the target RNA is attached at the site of engineered termini generated by circular permutation, whereas in the group II intron scaffold, it is inserted into an internal stem-loop. The latter design may provide more rigid structural coupling between the scaffold and the target RNA, contributing to the higher resolution reconstruction, although additional studies will be needed to determine the extent to which this contributes to reconstruction quality. The group II intron scaffold also offers practical advantages. Scaffolded RNAs can be purified under native conditions without posttranscriptional refolding, reducing the likelihood of misfolding and simplifying sample preparation. In addition, the group IIC intron largely eliminates the preferred particle orientation commonly observed for small RNAs in cryo-EM, resulting in more isotropic angular distributions (77).

## Perspectives and future directions

The functional landscape of RNA is remarkably diverse, despite the chemical similarity shared by all RNA molecules. As interest in understanding functional RNAs continues to grow, effective strategies for designing and synthesizing these molecules are increasingly important for structural and biochemical studies. A variety of stable RNA structures can serve as scaffolds for RNAs of interest, allowing researchers to select platforms best suited to specific applications. In this review, we have described several strategies for studying RNA structure and function. These approaches repurpose naturally occurring RNA structural motifs and processing pathways, including tetraloop motifs, tRNA, 5S rRNA, pRNA assembly, and group I and group II introns, to create versatile platforms. It is important to note that the resolution of a scaffolded target RNA depends strongly on the size and intrinsic structural rigidity of the target itself. Larger RNAs that adopt compact, well-defined architectures are generally more amenable to high-resolution structure determination, whereas smaller or more flexible RNAs may remain difficult to resolve even when attached to a well-ordered scaffold.

No single RNA scaffold is optimal for every application. Instead, scaffold selection should be guided by several considerations, including the method of structure determination, engineering flexibility, target RNA size and complexity, and ease of production. X-ray crystallography generally requires relatively large quantities of highly concentrated, homogeneous RNA, particularly for screening multiple crystallization conditions. In this context, tRNA and 5S rRNA scaffolds offer an advantage because they enable large-scale recombinant production of scaffolded RNAs in bacteria. These scaffolds may also facilitate crystallization by providing well-folded structural elements that can participate in crystal contacts. In contrast, cryo-EM typically requires substantially less sample but benefits from scaffolds that increase the effective size of the target RNA and provide robust features for particle identification and alignment. The pARIS and group I/II intron scaffolds are particularly well suited for this purpose. The rigidity of the junction between the scaffold and target RNA is another important consideration. Engineering the target RNA into a structurally constrained internal region of the scaffold can provide more rigid coupling than attachment at the termini and may therefore improve the resolution of the target RNA. This principle is exemplified by the pRNA^cp^ and group II intron scaffolds. In addition to scaffold selection, iterative optimization of the workflow, including optimization of sample and buffer conditions, grid preparation, data collection and analysis, is required for a successful RNA structure determination using cryo-EM (80). The major characteristics, advantages, and limitations of the RNA scaffolds discussed in this review are summarized in Table 2.

**Table 2:** Comparison of RNA-scaffold approaches

Feature	tRNA	5S rRNA	Φ29 pRNA (pARIS)	Group I intron	Group II intron
Structural application	X-ray; cryo-EM	X-ray; cryo-EM potential	cryo-EM	cryo-EM	cryo-EM
Scaffold size	∼70 nt	∼120 nt	∼71 nt pRNA assembled on Φ29 prohead	∼400 nt	∼400 nt
Target attachment site	anticodon stem (internal)	engineered loop B (internal)	pRNA (terminal) pRNA^cp^ (internal)	circularly permuted P6 (terminal)	Domain III (internal)
Demonstrated target size^a^	70–300 nt	40–75 nt	50–70 nt	50–70 nt	80–210 nt
Preferred target junction	continuous dsRNA stem	continuous dsRNA stem	continuous dsRNA stem	continuous dsRNA stem	continuous dsRNA stem
RNA production	recombinant expression in *E. coli;* purification by anion exchange chromatography; no refolding	recombinant expression in *E. coli;* purification by anion exchange chromatography; no refolding	*In vitro* transcription; purification by anion exchange chromatograph; no refolding	*In vitro* transcription; purification by denaturing PAGE; refolding prior to use	*In vitro* transcription; purification by diafiltration; no refolding
Key advantages	high-yield RNA; precise RNA ends	high-yield RNA; precise RNA ends	five copies of target RNA on a Φ29 prohead	large, rigid scaffold	large, rigid scaffold
Structural advantages	compact; promote crystallization; compatible with tetraloop	compact; compatible with tetraloop	particle picking and alignment; five pRNA copies enable symmetry expansion	particle picking and alignment	particle picking and alignment; favorable particle orientations
Requirements	stable expression of RNA chimera in *E. coli*	stable expression of RNA chimera in *E. coli*	Φ29 prohead production and assembly; focused refinement	refolding of large intron scaffold; may require focused refinement	proper folding of large intron scaffold; focused refinement
Limitations	small scaffold for cryo-EM	limited application in high-resolution structure determination	target resolution may be lower than the scaffold	target resolution may be lower than the scaffold	target resolution may be lower than the scaffold

a Representative demonstrated examples should not be interpreted as absolute size limits.

The RNA scaffolds discussed here are not exhaustive, and the development of additional RNA scaffolds remains an active area of research. Recent advances have extended the RNA scaffold toolbox by introducing engineered RNA assemblies specifically designed for structural biology applications. For example, symmetric RNA dimers of the Rous sarcoma virus frameshifting element have been developed as cryo-EM scaffolds. The two-fold symmetry increases the effective molecular weight of the target RNA and enables symmetry averaging during image processing, thereby improving particle alignment and reconstruction (81). Similarly, homo-oligomeric RNA rings have been generated by introducing complementary kissing-loop interactions into target RNA, allowing the self-assembly of higher-order RNA complexes suitable for cryo-EM analysis (82). Using these symmetric and multimeric RNA approaches, the structures of known RNA targets ranging from approximately 70 to 400 nt have been determined at resolutions of 3 to 5 Å.

Despite their structural diversity, most naturally derived RNA scaffolds share a common architectural constraint: the inserted RNA is typically presented as a stem-loop with base-paired 5′ and 3′ termini. While this strategy promotes structural stability and effective folding without affecting the scaffold RNA, it also limits the incorporation of RNAs that require free termini or long-range intramolecular interactions. Consequently, current scaffold approaches are well suited for hairpins, aptamers, and short structured motifs but are less adaptable to larger or more complex RNA architectures. Future scaffold designs may benefit from greater architectural flexibility, enabling the presentation of diverse RNA folds without imposing stem-loop constraints. As RNA structural biology, AI-guided RNA prediction, and RNA therapeutics continue to converge, next-generation RNA scaffolds will likely evolve into programmable platforms capable of controlling RNA folding, molecular recognition, and biological function. Such advances will expand our ability to interrogate RNA biology while enabling new applications in structural biology, therapeutics, and synthetic biology.

## Conflict of interest

The authors declare that they have no conflicts of interest with the contents of this article.
